# Prospective Evaluation of Ghrelin and Des-Acyl Ghrelin Plasma Levels in Children with Newly Diagnosed Epilepsy: Evidence for Reduced Ghrelin-to-Des-Acyl Ghrelin Ratio in Generalized Epilepsies

**DOI:** 10.3390/jpm12040527

**Published:** 2022-03-25

**Authors:** Anna-Maria Costa, Tommaso Lo Barco, Elisabetta Spezia, Valerio Conti, Laura Roli, Lorenza Marini, Sara Minghetti, Elisa Caramaschi, Laura Pietrangelo, Luca Pecoraro, Fabio D’Achille, Paola Accorsi, Tommaso Trenti, Federico Melani, Carla Marini, Renzo Guerrini, Francesca Darra, Patrizia Bergonzini, Giuseppe Biagini

**Affiliations:** 1 Department of Biomedical, Metabolic and Neural Sciences, University of Modena and Reggio Emilia, Via G. Campi 287, 41125 Modena, Italy; annamaria.costa@unimore.it; 2 Child Neuropsychiatry, Department of Surgical Sciences, Dentistry, Gynecology and Pediatrics, University of Verona, 37126 Verona, Italy; tommaso.lobarco@gmail.com (T.L.B.); francesca.darra@univr.it (F.D.); 3 PhD School of Clinical and Experimental Medicine (CEM), University of Modena and Reggio Emilia, Via G. Campi 287, 41125 Modena, Italy; 4 Pediatric Neurology Unit, Department of Mother and Child, University Hospital of Modena, 41125 Modena, Italy; spezia.elisabetta@aou.mo.it (E.S.); caramaschi.elisa@policlinico.mo.it (E.C.); bergonzini.patrizia@policlinico.mo.it (P.B.); 5 Pediatric Neurology, Neurogenetics, and Neurobiology Unit and Laboratories, A. Meyer Children’s Hospital, 50139 Florence, Italy; valerio.conti@meyer.it (V.C.); lorenza.marini@meyer.it (L.M.); sara.minghetti@hotmail.it (S.M.); laura.pietrangelo89@gmail.com (L.P.); federico.melani@meyer.it (F.M.); carla.marini@ospedaliriuniti.marche.it (C.M.); renzo.guerrini@meyer.it (R.G.); 6 Department of Laboratory Medicine and Pathology, Azienda USL, 41126 Modena, Italy; l.roli@ausl.mo.it (L.R.); f.dachille@ausl.mo.it (F.D.); t.trenti@ausl.mo.it (T.T.); 7 Paediatric Clinic, ASST Mantua, 46100 Mantua, Italy; luca.pecoraro@asst-mantova.it (L.P.); paola.accorsi@asst-mantova.it (P.A.)

**Keywords:** antiseizure medications, children, epilepsy, generalized epilepsy, ghrelin, plasma

## Abstract

Children with epilepsy and identified as responders to antiseizure medications (ASMs) were found to present markedly higher ghrelin plasma levels when compared to drug-resistant patients. However, it was undetermined if this phenotype could be influenced by the ASMs. Here, we prospectively investigated total ghrelin and des-acyl ghrelin (DAG) plasma levels by enzyme-linked immunosorbent assay before and after ASM administration. Inclusion criteria were: (i) subject with a suspicion of epilepsy; (ii) age ranging from 0 to 16 years; and (iii) informed consent signed by parents or caregivers. Exclusion criteria were acute or chronic metabolic disorders with occasional convulsions but without epilepsy. Fifty patients were followed over a period of one year in Italian neuropediatric centers. Apart from a few exceptions, the majority of children were responsive to ASMs. No differences were found in total ghrelin and DAG levels before and after the treatment, but total ghrelin levels were significantly lower in children with generalized epilepsy compared to those with combined focal and generalized epilepsy. Moreover, the ghrelin-to-DAG ratio was also markedly lower in generalized epilepsies compared to all the other types of epilepsy. Finally, ghrelin was unchanged by ASMs, including the first (e.g., carbamazepine), second (levetiracetam), and third (lacosamide) generation of anticonvulsants.

## 1. Introduction

Epilepsy is a neurological disorder characterized by spontaneous and recurrent seizures [1]. It affects approximately 50 million people, of which up to 30% suffer from pharmacoresistant epilepsy [2]. In this regard, an incomplete understanding of risk factors and molecular mechanisms of the disease along with a lack of reliable biomarkers are major limitations in the prompt identification of pharmacoresistant epilepsy [3,4]. Indeed, the advancement of knowledge on these issues could help in predicting who will develop refractoriness to antiseizure medications (ASMs) and may allow for a more efficient identification of candidates for novel drug therapies for epilepsy. In this regard, peptides have recently been under consideration as potential new targets [5].

Ghrelin, a gastric-mucosa-secreted neuropeptide hormone, is an endogenous ligand of the growth hormone secretagogues receptor 1a (GHS-R1a) [6]. In addition to its well-established function in the stimulation of growth hormone (GH) secretion and food intake [7,8,9,10], growing evidence suggests a key role of ghrelin in different physiological and pathological conditions, including epilepsy [5,11,12]. In particular, both ghrelin and des-acyl ghrelin (DAG), as well as ghrelin analogous, have been shown to possess anticonvulsant properties. Interestingly, we previously reported that ghrelin and the cognate peptide DAG were significantly higher in the plasma of children positively responding to ASMs (responders) [13], raising the question of whether these hormones could be increased by the administration of ASMs, or alternatively, the children committed to be responders to ASMs could be characterized by pre-existing higher ghrelin plasma levels [14].

To address this question, we designed a prospective, polycentric study to evaluate the plasma levels of total ghrelin, ghrelin, and DAG before ASM administration (T0), 2 months (T1), and 12 months (T2) after the onset of ASM treatment in children with epilepsy, defined according to their specific demographic and clinical features.

## 2. Materials and Methods

### 2.1. Experimental Design

**Figure 1:**
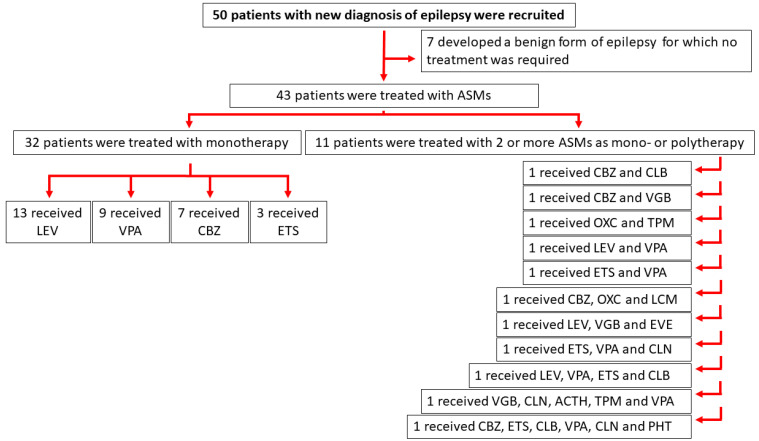
Flowchart illustrating the patients’ enrolment and treatment subsequent to diagnosis of epilepsy. Antiseizure medications (ASMs) were provided for therapeutic reasons by the pediatrician in charge. Precisely: (i) first generation of ASMs included adrenocorticotropic hormone (ACTH), carbamazepine (CBZ), clonazepam (CLN), ethosuximide (ETS), phenytoin (PHT), valproic acid (VPA); (ii) second generation of ASMs included levetiracetam (LEV), oxcarbazepine (OXC), topiramate (TPM), and vigabatrin (VGB); (iii) third generation of ASMs included clobazam (CLB), everolimus (EVE), and lacosamide (LCM).

We considered a cohort of 50 subjects composed of male and female patients with newly diagnosed epilepsy who were admitted to the neuropediatric hospitalist service in Modena (*n* = 21), Verona (*n* = 16), and Florence (*n* = 13). The project focused on pediatric epilepsy in coherence with previous studies [9,13,15,16] and took place between September 2019 and January 2022. The Ethics Committee approved the research protocol according to local regulations. The project did not involve the administration of ASMs, apart from those (e.g., valproate, levetiracetam, carbamazepine, topiramate, ethosuximide, phenobarbital, and vigabatrin) which were provided for therapeutic reasons by the pediatrician in charge. Thus, the alteration in plasma levels of total ghrelin and DAG were analyzed as a consequence of the normal protocol used in the administration of specific ASMs, disregarding the drug generation which each ASM belonged to (Figure 1).

The inclusion criteria were: (i) suspect of epilepsy; (ii) age ranging from 0 to 16 years; and (iii) informed consent signed by parents or caregivers. The exclusion criteria were acute or chronic metabolic diseases with occasional convulsions but without epilepsy. Demographic data, clinical features, diagnostic findings, and therapeutic interventions (i.e., age, gender, body weight, height, body mass index, day and cause of hospitalization, type of epilepsy, seizure frequency, etiology, comorbidity, drug responses and related adverse events) were acquired from patients by a common case report form to assure consistency.

### 2.2. Patients with Newly Diagnosed Epilepsy

The information about the pediatric patients’ demographic and clinical features enrolled in this study is illustrated in Table 1. Briefly, the median age of patients treated with at least one ASM was 7.9 years (interquartile range [IQR] 3.8 and 10.5); 22 were female (51%). These patients were divided according to the type of epilepsy (focal, generalized, combined generalized and focal or unknown). The etiology was assessed. Additionally, 7 out of 50 patients, who were excluded because they developed a benign form of epilepsy for which no treatment was required, had a median age of 6.7 years (IQR 4.0 and 8.5); 5 were female (71%).

### 2.3. Sample Processing

Fasting blood samples (8:00–9:00 a.m.) were obtained (3 mL) and coded to assure a blind processing for immunoassays. In this regard, blood samples were collected at a time in which ASMs were monitored (about 2 and 12 months) but also before starting therapy. Particularly, blood collected in tubes with dipotassium ethylenediaminetetraacetate dihydrate and 10% (*v/v*) protease inhibitor cocktail P2714 (Sigma Aldrich, Milan, Italy) was gently shaken, quickly placed on ice, and centrifuged for 15 min (1800× *g* at 4 °C) to store the plasma in sterile microtubes at −80 °C. Notably, 4-(2-aminoethyl) benzenesulphonyl fluoride hydrochloride, contained in P2714, was able to block the conversion of ghrelin to DAG, even when samples were kept frozen for months [17].

### 2.4. The Enzyme-Linked Immunosorbent Assay

The enzyme-linked immunosorbent assay (ELISA) kit for human ghrelin (total) (EZGRT-89K) was obtained from Merck Millipore (Milan, Italy). At variance, the human unacylated ghrelin ELISA kit (A05319) was provided by Bertin Pharma (Montigny-le-Bretonneux, Île-de-France, France). We also planned to use the ELISA kit for human ghrelin (active) (EZGRA-88K; Merck Millipore, Burlington, MA, USA). However, after an initial easy availability, during the COVID-19 pandemic, we found difficulties purchasing this ELISA Kit. For this reason, ghrelin plasma levels were calculated by the subtraction of DAG from total ghrelin. All immunoassays were performed according to the manufacturer specifications. For total ghrelin, the plate reader (DSX System, Dynex technologies, Chantilly, VA, USA) was used to read absorbance at 450 nm and 590 nm. The theoretical minimum detecting concentration of the assay was 50 pg/mL, whereas the intra- and inter-assay variability were ±1.26% and 7.81%, respectively. For DAG, the plate reader was used to read the plate at a wavelength between 405 and 414 nm. The limit of detection in the sample before the plasma dilution (1:5) was <10 pg/mL. The intra-assay coefficient of variation for DAG was 6.8%. For the same peptide, the inter-assay coefficient of variation was 12.5%.

### 2.5. Statistical Analysis

A power analysis was performed using G*power (version 3.1.9.2) [18] with an effect size of 0.82, α-error of 0.02, and a power (1-β) of 0.80, resulting in a sample size of 50 patients after correction for drop-outs. Data from immunoassays were analyzed using: (i) the Kruskal–Wallis one-way analysis of variance (ANOVA) on ranks, (ii) one-way ANOVA followed by Duncan’s test for multiple comparisons, (iii) two-way repeated measures ANOVA, and (iv) the Mann–Whitney rank sum test. Spearman correlation coefficients were used to model the relationships between variables. Outliers, identified by Grubbs’ test, were excluded. Statistical analyses were performed with SigmaPlot 13 (Systat Software). Data are represented as means with standard error of the mean (SEM) or median and IQR, and they were regarded significantly different at *p <* 0.05.

## 3. Results

### 3.1. Total Ghrelin and Ghrelin-to-DAG Ratio of Plasma Obtained at Different Time Points in Male and Female Pediatric Patients

The examined cohort of patients with newly diagnosed epilepsy was composed of 43 children treated with ASMs. The remaining patients were excluded from the analysis. Due to the COVID-19 pandemic or other impediments, 7 out of 43 patients did not take a blood sample at T1 and another 12 out of 43 patients did not take a blood sample at T2. In addition, 5 out of 43 patients left the study at T2. Moreover, 77% of children were seizure-free already at T1, while 16% of children continued to present seizures at T2, but only 11% seemed to display possible drug resistance. Four children (11%) were affected by focal (1), focal and generalized (2) or generalized (1) epilepsy. However, further investigation should be conducted because, according to the definition, patients with epilepsy might be classified as non-responders in presence of failure of at least 2 first-line ASMs, the occurrence of an average of 1 seizure per month for more than 18 months, and a lack of a more than 3-month seizure-free gap during the considered 18 months [19].

**Figure 2:**
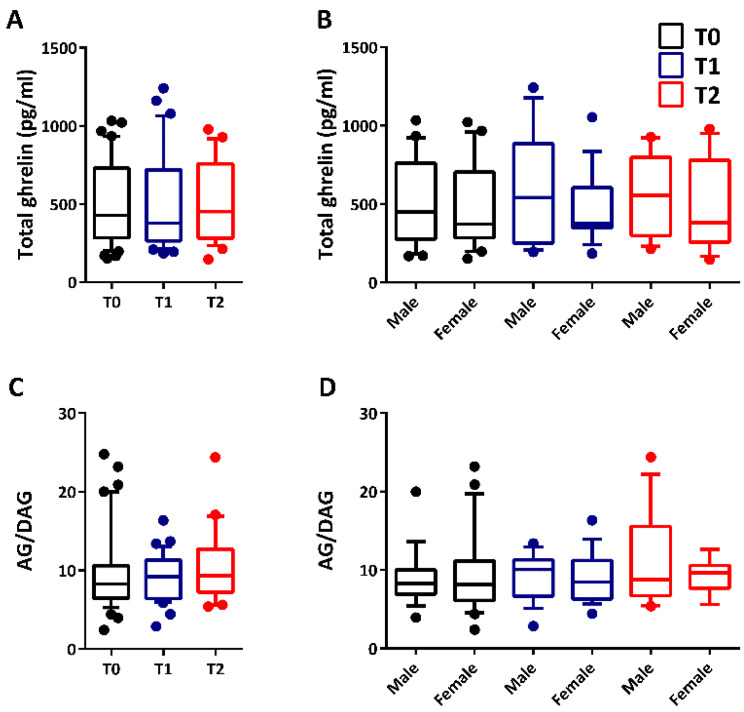
Total ghrelin and ghrelin (AG)-to-des-acyl ghrelin (DAG) ratio in plasma before and after drug treatment in male and female pediatric patients. In (**A**), no changes were reported for total ghrelin plasma levels after 2 months (T1; male, *n* = 18 and female, *n* = 18) or 12 months (T2; male, *n* = 13 and female, *n* = 13) from the onset of therapy (T0; male, *n* = 21 and female, *n* = 22). In (**B**), no differences were determined between male and female pediatric patients at the same time intervals. Similarly, no changes were observed for the ghrelin-to-DAG ratio based on time (**C**) and gender (**D**).

The median concentration of total ghrelin in plasma did not change (*p =* 0.903; Kruskal–Wallis test) in all patients before the administration of ASM (T0: 427.452 pg/mL; 282.814–730.335) and after 2 months (T1: 377.760 pg/mL; 263.865–717.535) or 12 months (T2: 451.048 pg/mL; 282.255–757.250) of ASM administration (Figure 2A). No differences (*p =* 0.956) were reported at the same time intervals when comparing males and females (Figure 2B).

Similarly, the median concentration of DAG in plasma was unchanged (*p =* 0.873) in all patients before treatment with ASMs (T0: 42.858 pg/mL; 25.656–71.322) and after 2 months (T1: 43.736 pg/mL; 31.918–63.391) or 12 months (T2: 40.222 pg/mL; 27.221–58.269) of drug therapy. We used the median concentration of DAG to calculate the ghrelin plasma levels by subtracting DAG from total ghrelin values. Then, the ghrelin plasma levels were used to determine the ghrelin-to-DAG ratio in plasma of all pediatric patients. In this regard, no changes in the ghrelin-to-DAG ratio (*p =* 0.540) were observed in plasma after drug treatment (Figure 2C). Additionally, no changes based on gender (*p =* 0.829) were reported in the case of the ghrelin-to-DAG ratio (Figure 2D).

### 3.2. Total Ghrelin and Ghrelin-to-DAG Ratio in Plasma of Children with Different Types of Epilepsy

**Figure 3:**
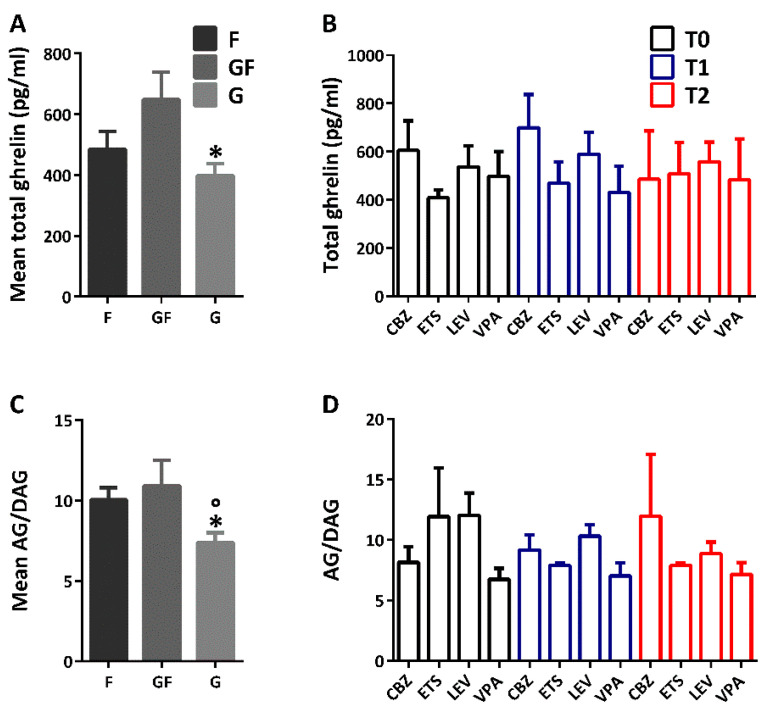
Total ghrelin and ghrelin (AG)-to-des-acyl ghrelin (DAG) ratio in plasma of children with different types of epilepsy and treated with appropriate antiseizure medications (ASMs). In (**A**), significantly low levels of mean total ghrelin plasma levels were reported in generalized epilepsies (G) in comparison to combined generalized and focal (GF) epilepsies. In (**B**), the mean total ghrelin in plasma at basal levels (CBZ, *n* = 7; ETS, *n* = 3; LEV, *n* = 13; VPA, *n* = 9) was not affected by the type of ASM at T1 (CBZ, *n* = 6; ETS, *n* = 2; LEV, *n* = 11; VPA, *n* = 8) and T2 (CBZ, *n* = 3; ETS, *n* = 2; LEV, *n* = 9; VPA, *n* = 4). Significantly low levels of AG-to-DAG ratio were reported only in children suffering from G, in comparison to focal (F) and GF epilepsies (**C**). These findings were also not affected by the time of ASM or time (**D**). * *p <* 0.050 G vs. GF; ° *p <* 0.050 G vs. F; Duncan’s method. Abbreviations: CBZ, carbamazepine; ETS, ethosuximide; LEV, levetiracetam; VPA, valproic acid.

The examined cohort of patients was composed of children with focal (*n* = 19), generalized (*n* = 16), or combined generalized and focal (*n* = 8) epilepsies. In comparison to children with combined generalized and focal epilepsy (total ghrelin: 648.159 ± 90.400 pg/mL), those affected by a generalized form of epilepsy had significantly lower levels of mean total ghrelin (397.806 ± 39.812 pg/mL; *p <* 0.050; Duncan’s method). Instead, no significant difference was determined between focal (total ghrelin: 485.001 ± 58.546 pg/mL) and combined generalized and focal epilepsy (Figure 3A). Furthermore, the total ghrelin plasma levels were analyzed according to the adopted ASMs. This analysis showed no statistically significant effect of both ASM (F(3,8) = 0.273, *p =* 0.843; two-way repeated measures ANOVA) and time (F(2,8) = 3.948, *p* = 0.118). Similarly, there was no statistically significant interaction between the type of ASM and time in all children (F(6,8) = 1.713, *p* = 0.235), indicating that all ASMs did not modify the total ghrelin plasma levels (Figure 3B).

The mean ghrelin-to-DAG ratio was also significantly lower in patients with generalized epilepsy (*p <* 0.050; Duncan’s method), in comparison to those affected by focal or combined generalized and focal epilepsies (Figure 3C). Also in this case, no statistically significant effects of both ASM (F(3,30) = 0.450, *p* = 0.719; two-way repeated measures ANOVA) and time (F(2,30) = 0.171, *p* = 0.843) were reported. Similarly, there was no statistically significant interaction between the type of ASM and time in all children (F(6,30) = 1.708, *p* = 0.153), indicating that ASMs did not modify the ghrelin-to-DAG ratio in plasma (Figure 3D).

The mean age of children with generalized epilepsies (9.354 ± 0.915 years) was significantly different (*p <* 0.050; Duncan’s method) from that of children with combined generalized and focal epilepsy (4.678 ± 1.202 years). Instead, no differences were reported between children with combined generalized and focal epilepsy and those with focal epilepsy (6.952 ± 0.920 years), as well as between this last phenotype and the children with generalized epilepsies. These findings suggest that the difference found between combined generalized and focal epilepsy and generalized epilepsy (Figure 3A) might be age-dependent.

### 3.3. Correlations between Total Ghrelin or Ghrelin-to-DAG Ratio and the Patient’s Characteristics

**Figure 4:**
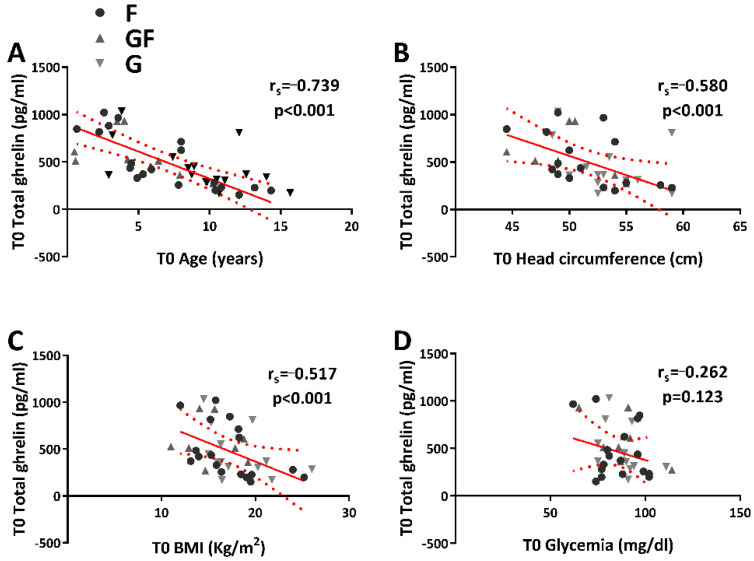
Relationship between total ghrelin plasma levels and age, head circumference, body mass index (BMI), and glycemia (**A**–**D**). Spearman correlation coefficients are reported.

Significant negative correlations (r_s_ = −0.739, *p <* 0.001) were determined between total ghrelin plasma levels and age (Figure 4A). The total ghrelin plasma levels were also negatively correlated (Figure 4B, C) with head circumference (r_s_ = −0.580, *p <* 0.001) and BMI (r_s_ = −0.517, *p <* 0.001). At variance, no correlation (r_s_ = −0.262, *p =* 0.123) was observed between total ghrelin plasma levels and glycemia (Figure 4D).

**Figure 5:**
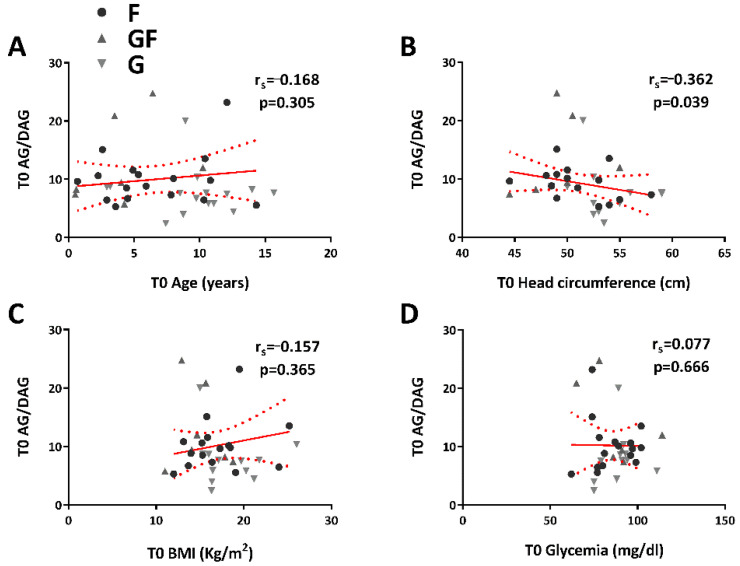
Relationship between ghrelin (AG)-to-des-acyl ghrelin (DAG) ratio and age, head circumference, body mass index (BMI), and glycemia (**A**–**D**). Spearman correlation coefficients are reported.

Furthermore, no correlation (r_s_ = −0.168, *p =* 0.305) was defined between the ghrelin-to-DAG ratio and age (Figure 5A), but it was instead present between the ghrelin-to-DAG ratio (Figure 5B) and head circumference (r_s_ = −0.362, *p =* 0.039). The ghrelin-to-DAG ratio (Figure 5C, D) was not correlated to BMI (r_s_ = −0.157, *p =* 0.365) or glycemia (r_s_ = 0.077, *p =* 0.666).

### 3.4. Comorbidities, Total Ghrelin, and Ghrelin-to-DAG Ratio in Plasma of Children with Newly Diagnosed Epilepsy

**Figure 6:**
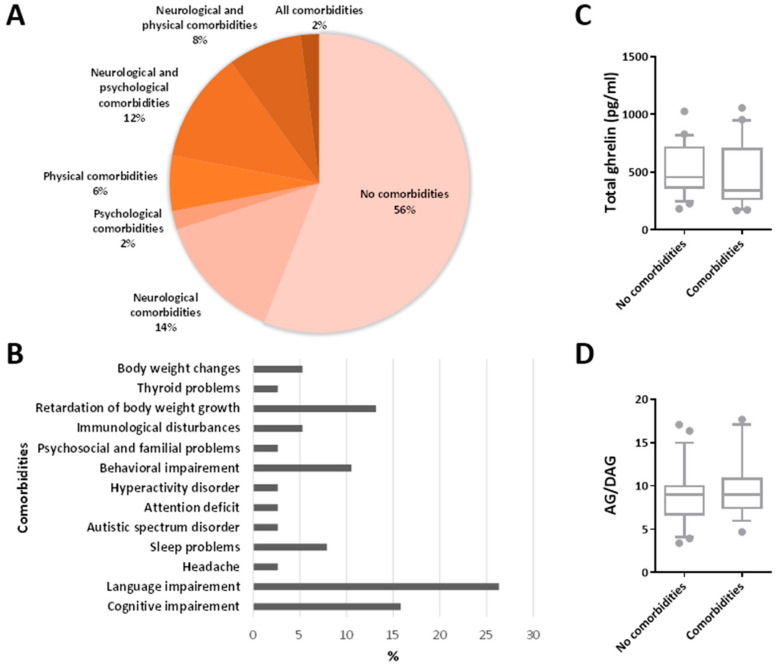
Total ghrelin and ghrelin (AG)-to-des-acyl ghrelin (DAG) ratio in plasma and comorbidities. In (**A**), the percentage of patients displaying comorbidities is reported. In (**B**), the prevalence of each type of comorbidity in the examined cohort of patients is represented in percentage. In comparison to children without comorbidities, the total ghrelin plasma levels and AG-to-DAG ratio were not significantly changed in children with newly diagnosed epilepsy, displaying comorbidities (**C**,**D**).

As previously suggested [20], comorbidities of childhood epilepsy could be divided into three categories: neurological, psychological, and physical comorbidities. The examined cohort of patients was composed of children with no comorbidities (56%) or at least one category of comorbidity: (i) neurological comorbidities (14%); (ii) psychological comorbidities (2%); and physical comorbidities (6%). Some children had two different categories of comorbidity: (i) neurological and psychological comorbidities (12%) and (ii) neurological and physical comorbidities (8%). Only 2% of patients displayed all comorbidities (Figure 6A).

The prevalence of each type of comorbidity was calculated in percentage and divided by the category (Figure 6B). Specifically, the neurological comorbidities reported in children were: (i) cognitive impairment (16%); (ii) language impairment (26%); (iii) headache (3%); and iv) sleep problems (8%). Additionally, the most common psychological comorbidities were: (i) autistic spectrum disorder (3%); (ii) attention deficit (3%); (iii) hyperactivity disorder (3%); (iv) behavioral impairment (11%); and (v) psychosocial and familial problems (3%). Among the physical comorbidities, the most common were: (i) immunological disturbances (5%); (ii) retardation of the body weight growth (13%); (iii) thyroid problems (3%); and (iv) body weight changes (5%).

No differences in the median concentration of the total ghrelin in plasma (*p =* 0.301; Mann–Whitney rank sum test) were found between children with comorbidities (341.429 pg/mL; 266.223–701.517) or without comorbidities (454.920 pg/mL; 365.452–711.010) (Figure 6C). Similarly, the median ghrelin-to-DAG ratio was not significantly different (*p* = 0.608) between children with or without comorbidities (Figure 6D).

## 4. Discussion

This study aimed to clarify whether plasma levels of the neuroactive peptide ghrelin could vary in response to treatment with different ASMs [14]. Our study clearly demonstrated that the therapeutic treatment could not modify plasma levels of total ghrelin and DAG, as measured in seizure-free periods, and thus the higher plasma levels for ghrelin and DAG found in responders to ASMs [13] was probably already present in patients committed to a better response to ASMs. Then, we found that children displaying focal or combined generalized and focal epilepsy had a higher ghrelin-to-DAG ratio in comparison to those affected by generalized epilepsy. In addition to this, other features, such as age, head circumference, and BMI, seemed to be related to the plasma levels of total ghrelin.

In the literature, other studies investigated ghrelin levels during the seizure-free interictal periods in relation to the disease progression, but the analyzed isoform was undefined in all studies [21,22,23,24,25], making the results’ interpretation difficult [5]. In general, most of the above-cited studies support our data, as they did not find significant differences between the treatment and pre-treatment periods [21,22,23,25]. For instance, no changes in serum levels of glucose, insulin, cortisol, leptin, neuropeptide Y, galanin, and ghrelin were observed before the treatment with oxcarbazepine and 6–18 months later in pediatric patients with an age range of 3.0–16.4 years [21]. In comparison to the pre-treatment period, no change in the plasma ghrelin and adiponectin levels were found at 6–12 months in 20 prepubertal children (6–12 years) with idiopathic epilepsy treated with valproic acid. Instead, these authors reported a weight gain occurring during the administration of valproic acid which was related to an increase in levels of insulin, leptin, and neuropeptide Y [22]. In another study, a significant decrease in the mean BMI was found in 20 prepubertal children (4–12 years) with epilepsy without any significant change in serum glucose, ghrelin, neuropeptide Y, or insulin levels at the third and six months of the administration of topiramate compared to pre-treatment levels. In the same study, the changes in the mean BMI were related to a reduction in the fasting insulin-to-glucose ratio and serum cortisol and leptin levels [23]. An additional prospective, case–control comparative study reported that no significant changes were present in the ghrelin serum levels of children (6–10 years) with epilepsy receiving levetiracetam as monotherapy for 6 months [25]. However, in some cases, the ghrelin levels seemed to increase after drug treatment. For instance, one study described significant changes in ghrelin serum levels after initiation of treatment with ASMs [24]. Indeed, a significant decrease in ghrelin levels was reported in pubertal children after the treatment with valproic acid but not in pre-pubertal children receiving the same ASM or oxcarbazepine [24]. A further study demonstrated that the serum, urine, and saliva levels of ghrelin and DAG were significantly lower 6 h after a seizure in pediatric patients not yet receiving treatment in comparison to 3 months after drug treatment and in healthy controls [26].

These discrepancies could be due to confounding factors such as an insufficient handling of samples to effectively block the conversion of ghrelin to DAG, as well as the use of a specific ASM to suppress the seizures [5]. Unlike many of the above-mentioned studies [21,22,23,24,25], our cohort was characterized by a large variety of ASMs to rule out the influence of a particular activity of the administered ASM. In addition to time and gender factors, we demonstrated that no changes in the total ghrelin plasma levels and the ghrelin-to-DAG ratio were related to a specific ASM. At variance, differences in the total ghrelin plasma levels were determined by the type of epilepsy and correlated with age, head circumference, and BMI, as already shown in the healthy controls by others [27]. Moreover, the ghrelin-to-DAG ratio was reduced only in generalized epilepsies in comparison to focal and combined generalized and focal epilepsies. To our knowledge, this is the first time that the ghrelin-to-DAG ratio has been found to be specifically affected in children with generalized epilepsy. Indeed, similar results were reported only in adult patients with a history of generalized seizures [28,29,30] by some, but not all, investigators [31,32] and, especially, no data were available before the drug treatment [5].

Seizures are defined as generalized when abnormal neuronal activity begins in a widespread distribution over both hemispheres [1,33]. However, some genetic generalized epilepsy syndromes, starting during childhood or adolescence and in some cases lasting into adulthood, might involve specific brain regions and mainly result from dysfunction of bilateral frontothalamocortical networks [34]. Indeed, patients with genetic generalized epilepsy syndromes seemed to display network changes in the prefrontal cortex and precuneus that should have triggered generalized spike wave discharges via increased connectivity within sensorimotor regions before the beginning of the discharges [35]. In general, the brain regions that are involved in generalized spike wave discharges mostly overlap with the default mode network, which is associated with the brain’s intrinsic activity and efficacious cognitive processes through its interplay with executive and attentional networks during goal-directed tasks [36]. Interestingly, it was suggested that network changes might be syndrome-specific, and thus patterns of altered default mode network connectivity in genetic generalized epilepsy syndromes should differ from those in focal epilepsies [37,38]. Among the genetic generalized epilepsy syndromes, childhood absence epilepsy could occasionally become refractory [39,40] to standard treatments and might be related to a reduced functional connectivity between frontomesial regions and the anterior insula and/or frontal operculum [41]. At variance, another genetic generalized epilepsy syndrome, known as juvenile myoclonic epilepsy, could be associated with a reduced connectivity within the prefrontal cognitive networks that were supposed to be responsible of working memory difficulties during working memory tasks [42,43].

Interestingly, some of the above-mentioned neocortical regions are able to modulate plasma levels of ghrelin in response to specific stimuli, and vice versa, these same regions seem to display a site-specific neuronal activation in response to changes in ghrelin levels. Specifically, ghrelin plasma levels were positively correlated with the activation of the prefrontal cortex, amygdala, and insula and negatively correlated with activation in subcortical areas such as the hypothalamus [44]. Ghrelin was shown to affect brain regions controlling rewards (e.g., striatum and prefrontal cortex), and ghrelin deficiency was shown to be related to disrupted reward-related brain activity in obesity [45]. Furthermore, it was suggested that structural changes in the brain regions implicated in executive control and self-referential processing occurred after bariatric surgery in obese patients, and these changes were accompanied by a reduction in fasting plasma ghrelin [46]. These findings imply the existence of a bi-directional relationship between the cerebral activity exerted by the frontal lobe and the ghrelin production that could have been disturbed by the epileptic activity developed during seizures occurring in our children with generalized epilepsy.

Overall, this study shows a lower plasma level of the ghrelin-to-DAG ratio only in children with generalized epilepsies. Nevertheless, the study has some limitations, mainly related to the fact that it was not possible to compare the various syndromes related to generalized epilepsy and specific comorbidities, such as cognitive impairments. In the future, it will be necessary to increase the number of patients with generalized epilepsy and comorbidity to make the results much more reliable. On the other hand, the study could offer an additional key to understanding the relationship between ghrelin and epilepsy.

## 5. Conclusions

Overall, our findings suggest that total ghrelin plasma levels and the ghrelin-to-DAG ratio were unchanged by ASMs, but they were determined by demographic and clinical features such as the type of epilepsy, age, head circumference, and BMI.

## Figures and Tables

**Table 1:** Demographic and clinical features of the examined cohort of patients.

	Patients Treated with ASMs	Untreated Patients
Female, *n* (% of group)	22 (51)	5 (71)
Male, *n* (% of group)	21 (49)	2 (29)
Age, y, median (IQR)	7.9 (3.8–10.5)	6.7 (4.0–8.5)
**Epilepsy types, *n* (% of group)**		
Focal	19 (44)	7 (100)
Generalized and focal	8 (19)	0 (0)
Generalized	16 (37)	0 (0)
Unknown	0 (0)	0 (0)
**Etiology classification, *n* (% of group)**		
Genetic	28 (65)	7 (100)
Structural	4 (9)	0 (0)
Metabolic	0 (0)	0 (0)
Immune	0 (0)	0 (0)
Infectious	0 (0)	0 (0)
Unknown	11 (26)	0 (0)

Abbreviations: ASM = antiseizure medication; IQR = interquartile range.

## Data Availability

The data presented in this study are available on request from the corresponding authors.
